# Comparative venom gland transcriptome surveys of the saw-scaled vipers (Viperidae: *Echis*) reveal substantial intra-family gene diversity and novel venom transcripts

**DOI:** 10.1186/1471-2164-10-564

**Published:** 2009-11-30

**Authors:** Nicholas R Casewell, Robert A Harrison, Wolfgang Wüster, Simon C Wagstaff

**Affiliations:** 1 School of Biological Sciences, Bangor University, Environment Centre Wales, Bangor, UK; 2 Alistair Reid Venom Research Unit, Liverpool School of Tropical Medicine, Liverpool, UK

## Abstract

**Background:**

Venom variation occurs at all taxonomical levels and can impact significantly upon the clinical manifestations and efficacy of antivenom therapy following snakebite. Variation in snake venom composition is thought to be subject to strong natural selection as a result of adaptation towards specific diets. Members of the medically important genus *Echis *exhibit considerable variation in venom composition, which has been demonstrated to co-evolve with evolutionary shifts in diet. We adopt a venom gland transcriptome approach in order to investigate the diversity of toxins in the genus and elucidate the mechanisms which result in prey-specific adaptations of venom composition.

**Results:**

Venom gland transcriptomes were created for *E. pyramidum leakeyi*, *E. coloratus *and *E. carinatus sochureki *by sequencing ~1000 expressed sequence tags from venom gland cDNA libraries. A standardised methodology allowed a comprehensive intra-genus comparison of the venom gland profiles to be undertaken, including the previously described *E. ocellatus *transcriptome. Blast annotation revealed the presence of snake venom metalloproteinases, C-type lectins, group II phopholipases A_2_, serine proteases, L-amino oxidases and growth factors in all transcriptomes throughout the genus. Transcripts encoding disintegrins, cysteine-rich secretory proteins and hyaluronidases were obtained from at least one, but not all, species. A representative group of novel venom transcripts exhibiting similarity to lysosomal acid lipase were identified from the *E. coloratus *transcriptome, whilst novel metallopeptidases exhibiting similarity to neprilysin and dipeptidyl peptidase III were identified from *E. p. leakeyi *and *E. coloratus *respectively.

**Conclusion:**

The comparison of *Echis *venom gland transcriptomes revealed substantial intrageneric venom variation in representations and cluster numbers of the most abundant venom toxin families. The expression profiles of established toxin groups exhibit little obvious association with venom-related adaptations to diet described from this genus. We suggest therefore that alterations in isoform diversity or transcript expression levels within the major venom protein families are likely to be responsible for prey specificity, rather than differences in the representation of entire toxin families or the recruitment of novel toxin families, although the recruitment of lysosomal acid lipase as a response to vertebrate feeding cannot be excluded. Evidence of marked intrageneric venom variation within the medically important genus *Echis *strongly advocates further investigations into the medical significance of venom variation in this genus and its impact upon antivenom therapy.

## Background

Snake venoms contain a complex mix of components, with biologically active proteins and peptides comprising the vast majority [1]. Variation in the composition of venom occurs at several taxonomical levels in multiple snake lineages [reviewed in [2,3]]. The view that variation in venom composition evolves primarily through neutral evolutionary processes [4-6] is not supported by other reports that snake venom composition is subject to strong natural selection as a result of adaptation towards specific diets [e.g. [7-10]]. Since the primary role of venom is to aid prey capture [2], it is perhaps unsurprising that variation in the protein composition of venom has been associated with significant dietary shifts in a number of genera [9-12]. Irrespective of the evolutionary forces underpinning venom protein composition, variation in venom components can significantly impact upon the clinical manifestations of snake envenoming [13-15] and, because the clinical efficacy of an antivenom may be largely restricted to the venom used in its manufacture, the success of antivenom therapy [16-18].

Envenoming by saw-scaled viper (Viperidae: *Echis*) species is thought to be responsible for more snakebite deaths worldwide than any other snake genus [19]. Envenomed victims typically suffer a combination of systemic and local haemorrhagic symptomatologies and up to 20% mortality rates without antivenom treatment [19-21]. Whilst the clinical symptoms are largely consistent throughout this widely distributed genus [20], cases of incomplete intrageneric antivenom efficacy have been documented, implying substantial inter-species venom variation [18,22-24]. We demonstrated that the four species complexes making up this genus, the *E. carinatus*, *E. ocellatus, E. pyramidum *and *E. coloratus *species groups [10,25], exhibit considerable vertebrate or invertebrate dietary preferences, *E. coloratus *being a vertebrate specialist whereas invertebrates feature prominently in the diet of the others. Since the proportions of consumed invertebrates correlated strongly with alterations in venom toxicity to scorpions, we believe the toxicity of the venom from these species to have co-evolved alongside evolutionary shifts in diet [10]. A preliminary venom protein analysis using reduced SDS-PAGE failed to identify an obvious link between venom composition and diet [10], justifying the use of a more comprehensive venom composition analysis in order to elucidate the mechanisms driving venom adaptations within the *Echis *viper genus.

Based on our earlier work with *E. ocellatus *[26], a comparative venom gland transcriptome approach was elected and we generated venom gland cDNA libraries from *E. coloratus*, *E. pyramidum leakeyi *and *E. carinatus sochureki*. Together with the existing *E. ocellatus *database, these provided DNA sequence data representing the venom gland transcriptomes for each of the four major species groups within the genus. The production of multiple *Echis *venom gland expressed sequence tag databases (vgDbEST) provides an unbiased overview of the transcriptional activity during venom synthesis in the venom glands of four species in this genus. This, the first comprehensive compilation of venom gland transcriptomes of congeneric snake species, was then interrogated to determine whether the mechanisms resulting in prey-specific adaptation of venom composition involve (i) the recruitment of novel prey-specific venom toxin transcripts, (ii) major changes in the expression levels of established toxin families, (iii) the diversification of functional isoforms within established toxin families or (iv) a combination of these factors.

## Results

EST data provides a powerful insight into the transcriptional activity of a tissue at a particular time point. Our protocols for the generation of venom gland EST databases provide a snapshot of transcriptional activity in the venom gland 3 days after venom expulsion, when transcription peaks [27] in preparation for new venom synthesis. Although each individual venom transcript cannot be correlated with the mature venom proteome without considerable extra experimental verification, our own work with *E. ocellatus *[28] shows there is a good general accordance between the venom proteome and that predicted from the venom gland transcriptome. Thus, whilst a cautionary approach is required when interpreting a correlation between transcriptome and proteome, the sensitivity and unbiased nature of venom gland transcriptome surveys can be valuable in the identification of rare, unusual or potentially novel toxins and their isoforms that are difficult to detect in the proteome [29].

**Figure 1:**
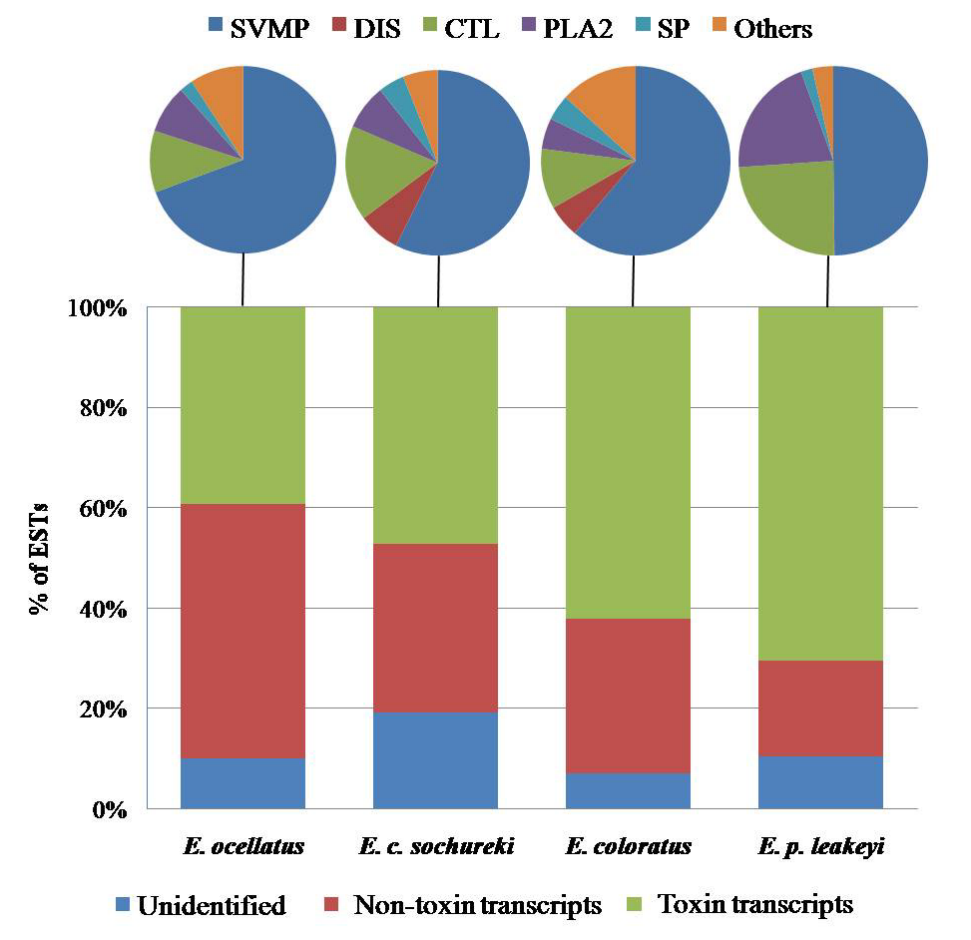
**The relative expression of annotated venom gland transcriptomes from four members of the genus *Echis***. Bar charts represent the proportions of BLAST-annotated ESTs; unidentified = non-significant hits. Toxin encoding transcripts are expanded as pie charts illustrating the proportional representation of snake venom metalloproteinases (SVMP), short coding disintegrins (DIS), C-type lectins (CTL), group II phospholipases A_2 _(PLA_2_), serine proteases (SP) and other less represented venom toxins (Others) in the transcriptomes of each *Echis *species

**Figure 2:**
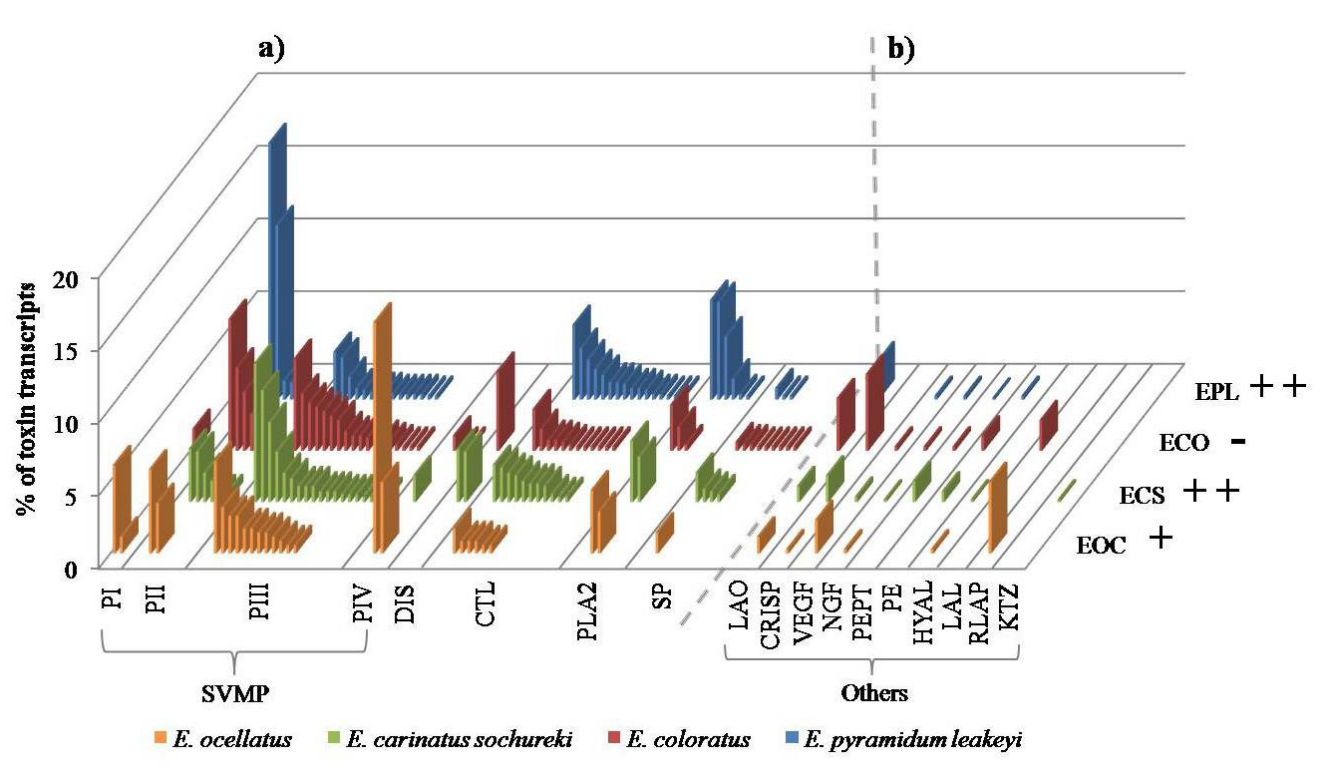
**The relative abundance and diversity of each *Echis *genus venom toxin family**. **a) **Relative expression levels of non-singleton clusters of the most representative venom toxin families and **b) **Relative expression levels of total non-singleton clusters and singletons representing the less numerically represented venom toxin families (Others) are expressed as a percentage of total toxin encoding transcripts. Column to the right indicates the proportion of invertebrate prey consumed and the corresponding correlation of venom toxicity to scorpions: ++, high; +, moderate; -, low [adapted from [10]]. Key - PI-PIV: sub-classes of snake venom metalloproteinases (SVMP); DIS: short coding disintegrins; CTL: C-type lectins; PLA2: group II phospholipases A_2_; SP: serine proteases; LAO: L-amino oxidases; CRISP: cysteine-rich secretory proteins; VEGF: vascular endothelial growth factors; NGF: nerve growth factors; PEPT: peptidases - aminopeptidase, dipeptidyl peptidase III and neprilysin; PE: Purine liberators - phosphdiesterase, 5'-nucleotidase and ectonucleoside triphosphate diphosphohydrolase (E-NTPase); HYAL: hyaluronidases; LAL: lysosomal acid lipases; RLAP: renin-like aspartic proteases; KTZ: kunitz-type protease inhibitors.

To provide a representative overview of the transcriptional variation in venom components in each species, whilst minimising compositional bias arising from intraspecific variation in venom composition, venom gland cDNA libraries were based on ten specimens of variable size and gender. Generated ESTs were clustered under high stringency conditions to assemble overlapping single sequence reads into full length gene objects where possible. Using BLAST, 80-93% of gene objects for each library were assigned a functional annotation based upon significant (>1e-05) scores against multiple databases. The majority of annotated ESTs (61-74%) were assigned to clusters representing distinct gene objects (additional file 1). The proportion of toxin encoding transcripts (enzymes and non-enzymatic toxins) assigned by BLAST homology, was typically greater than those encoding non-toxin transcripts (for example, those involved in cellular biosynthetic processes) and unidentified components (i.e. with no significant hit against the databases) (Figure 1). There were twice the numbers of unidentified ESTs in the *E. c. sochureki *vgDbESTs than in any of the other *Echis *vgDbESTs. As the bulk of these unidentified ESTs were singletons, not clustered gene objects, we interpret this to result from increases in unidentified 3' untranslated regions rather than unidentified novel toxin transcripts. The annotated venom toxin encoding profiles for the four *Echis *species revealed substantial variation in (i) the inferred expression levels and (ii) the cluster diversity within many toxin families (Figure 2, additional file 2). The details and potential implications of this species-specific variation in the representation of each toxin family will be discussed in turn.

### Snake venom metalloproteinases (SVMP)

The SVMP transcripts were the most abundant and divergent (in terms of cluster numbers) *Echis *venom toxin family (Figure 2) and comprised roughly half of the total toxin transcripts (Figure 1). The SVMPs are a diverse group of enzymes classified into those comprising only the metalloproteinase domain (PI) and those sequentially extended by a disintegrin domain (PII), a disintegrin-like and cysteine-rich domain (PIII) and the latter co-valently linked to C-type lectin-like components (PIV) [30]. Known and suspected modifications in domain structure are thought to account for the wide range of SVMP pathological activities, including haemorrhage, coagulopathy, fibrinolysis and prothrombin activation [30-32].

There were more PIII SVMP clusters in the genus *Echis *than any other toxin family clusters. The presence of apparent, extensive PIII SVMP gene diversification hints that evolutionary pressures are acting to increase the functional diversity of this SVMP group, highlighting their fundamental biological importance to the genus. In contrast, PI SVMP transcripts were present, albeit at low levels, only in the *E. coloratus *and *E. ocellatus *vgDbESTs. While the diversity of the PII SVMPs was substantially lower than that of the PIII SVMPs, their abundance differed between species. Thus, 80% of total *E. p. leakeyi *SVMP transcripts were PIIs (cluster EPL00005 comprised 38% of all SVMPs) and, although less numerically significant, 38% of the *E. coloratus *SVMPs were also PIIs. Despite intrageneric variation in abundance and diversity, analysis of PII contiguous sequences throughout the genus revealed the ubiquitous representation of motifs (RGD, KGD and VGD) involved in binding to the α_IIb_β_3_, α_v_β_3 _and α_5_β_1_integrins implicated in platelet aggregation inhibition [33,34]. The RGD-only representation of *E. p. leakeyi *PII SVMPs implies evolutionary conservation of this particular disintegrin motif, in contrast to the gene diversification observed in the PIIIs. We assigned some PIII SVMP transcripts as putative PIV SVMPs according to the presence of an additional cysteine residue in the cysteine-rich region at positions 397 or 400 [[28,30] (numbering from 30)]. These transcripts also form strongly supported monophyletic groups (data not shown) with homologues of SVMP PIVs previously characterised from venom proteomes; two of the three putative *E. coloratus *PIVs (ECO00075 & ECO00144) show the greatest sequence similarity to PIV SVMPs characterised from *Macrovipera lebetina *and *Daboia russelii *respectively [UniProt:Q7T046 and Q7LZ61], whereas all other *Echis *PIVs showed greatest similarity to the previously characterised *E. ocellatus *PIV SVMP, EOC00024 [28]. The relative representation of these putative PIV SVMPs was substantially greater in *E. ocellatus *(EOC00024 - 23% and EOC00022 -7%) than *E. coloratus *and *E. c. sochureki *(<4%); no PIV SVMPs were found in the *E. p. leakeyi *vgDbEST. Taken together, this implies that two divergent forms of PIV SVMPs may be uniquely present in *E. coloratus*, despite their low representation in this species.

We (SCW, RAH) recently identified a new *E. ocellatus *cDNA precursor encoding numerous QKW tripeptides and a polyH/G peptide that have potent SVMP-inhibiting activities [35]. Representatives of this SVMP inhibitory transcript were identified in each *Echis *vgDbEST (data not shown), but no correlation was identified between the proportional representation of the *Echis *SVMPs and their SVMP inhibitory transcripts.

### Disintegrins

Snake venom disintegrins are derived either from proteolytic processing of PII SVMP precursors [36] or are encoded by discreet PII-derived disintegrin-only genes, containing only a signal peptide and a disintegrin domain - previously described as 'short coding' disintegrins [37,38]. Representation of short coding disintegrins in the *Echis *genus is variable; small clusters were found in *E. c. sochureki *(4% and 3% of toxin transcripts) and *E. coloratus *(5%), whilst only a singleton transcript was found in *E. p. leakeyi*. Despite not being represented in the original *E. ocellatus *vgDbEST, we previously identified, by PCR, a sequence encoding the short coding disintegrin ocellatusin from this species [39], confirming the presence of short coding disintegrin transcripts throughout the *Echis *genus.

### C-type lectins (CTL)

The CTLs proved to be the next most abundant and diverse (by cluster numbers) group of *Echis *venom toxin encoding transcripts. As argued for the SVMPs, the substantial CTL cluster diversity and implied functional diversity would be consistent with the known variation in CTL activity. Thus, CTL isoforms typically act synergistically as homologous or heterologous multimers to promote or inhibit platelet aggregation and/or target distinct elements of the coagulation cascade [see [40,41]]. Each of the *Echis *species showed considerable CTL diversity (10-24% toxin encoding transcripts), with *E. p. leakeyi *exhibiting both the largest number of ESTs and cluster-diversity. Notably, clusters showing similarity to echicetin α and β, a platelet aggregation-inhibitor isolated from *E. c. sochureki *[42,43], were found throughout the *Echis *genus and are the most represented CTLs in both *E. c. sochureki *and *E. p. leakeyi*. Recently, *E. ocellatus *echicetin-like CTLs were demonstrated to be associated with forming the quaternary structure of PIV *E. ocellatus *SVMPs [28]. However, PIV SVMPs are absent from the *E. p. leakeyi *vgDbEST and present in only small numbers in *E. c. sochureki *(2%), implying that PIV-related binding may not be the sole function of echicetin. In contrast, each of the *Echis *vgDbESTs (except for *E. p. leakeyi*) contained clusters showing high sequence similarity to another PIV-related CTL, Factor X activator light chain 2 from *M. lebetina *[44], producing an *Echis *representational profile of CTLs matching that of the PIV SVMPs.

### Phospholipase A_2 _(PLA_2_)

Group II PLA_2_s are ubiquitously expressed in *Echis *species [45]. *Echis *PLA_2_s have been demonstrated to inhibit platelet aggregation and induce oedema, neurotoxicity and myotoxicity through multiple isoforms exhibiting high (Asp^49^) and low (Ser^49^) enzymatic activity [46-49]. Despite low representation and diversity in *E. coloratus*, *E. ocellatus *and *E. c. sochureki *(5-8% of toxin transcripts), an increase in representation (21%) and cluster diversity was observed in *E. p. leakeyi*, suggesting an important role for PLA_2 _activity in the venom of this species. Furthermore, both enzymatic PLA_2 _variants are conserved throughout the genus, highlighting the apparent importance of these functionally-distinct isoforms - presumably for prey capture. Given that Ser^49 ^PLA_2_s have only been isolated from the genera *Vipera *[50] and *Echis *[49], which are not sister taxa [51], we would expect the presence of this isoform in other members of the Viperinae. However, considering the absence of Ser^49 ^PLA_2_s from a *Bitis gabonica *vgDbEST [38], we cannot rule out convergent evolution of this myotoxic PLA_2 _type and its consequent functional importance in these genera.

### Serine proteases (SP)

The snake venom serine proteases are a multi-gene enzyme family acting upon platelet aggregation, blood coagulation and fibrinolytic pathways [reviewed in [41]]. Considering the severe coagulopathy observed in victims of *Echis *envenoming [19,31], the SPs are represented in amounts lower than predicted (2-5% of toxin encoding transcripts), particularly given their high representation in other, albeit distantly related, Viperidae species [52,53]. Interestingly, variations in cluster diversity are considerable, with nine clusters of low representation identified in *E. coloratus *compared to one in *E. ocellatus*. Despite low levels of representation, the unique variation in cluster diversity observed in *E. coloratus *implies multiple gene duplication events within this lineage; a process that underpins functional diversification in multi-gene venom proteins [8,54].

### L-amino oxidases (LAO)

Snake venom LAOs have been demonstrated to induce apoptosis and inhibit platelet function [reviewed in [55]]. While the mechanisms for these actions remain predominately uncharacterised, it seems clear that, unlike other snake venom toxin families, isoform diversity is not a requirement. Thus, the low representation (1-4% of toxin transcripts) observed in the *Echis *vgDbESTs is consistent with other viperid venom gland transcriptomes [26,38,52,53,56-59]. Indeed, the atypically high level of sequence conservation between all the *Echis *LAOs and those from other viperid genera (>80%) implies a conserved mechanism of action, whereby evolutionary pressures act to constrain diversification.

### Cysteine-rich secretory proteins (CRISP)

Members of the snake venom CRISP family interact with ion channels and exhibit the potential to block arterial smooth muscle contraction and nicotinic acetylcholine receptors [e.g. [60,61]]. The relative CRISP expression profiles vary considerably in the genus *Echis*, ranging from 5% of toxin encoding transcripts in *E. coloratus*, less than 2% in *E. c. sochureki *and *E. ocellatus *and none in *E. p. leakeyi*. Given that CRISPs are typically underrepresented toxin transcripts in Viperidae vgDbESTs [26,38,52,56-59], the abundant representation observed in *E. coloratus *implies an unidentified evolutionary pressure favouring transcriptional expression in this species. Its potential biological significance is further highlighted by the apparent absence of these toxins in the transcriptome of the most closely related species, *E. p. leakeyi*, which differs strongly in diet from *E. coloratus *[10].

### Other toxin components

Clusters encoding vascular endothelial growth factors and nerve growth factors were identified in small numbers (additional file 2) throughout the genus and, like the LAOs, each showed a high degree of sequence conservation. Similarly, and consistent with previous reports [62], the sequence homology of the new hyaluronidase singleton ESTs of *E. c. sochureki *and *E. ocellatus *was also considerable, and extended to hylauronidase sequences of other genera. It is apparent that evolutionary forces exist to conserve the sequence of this group of venom proteins, presumably because their role in disseminating venom toxins by reducing the viscosity of the extracellular matrix [29] is a universal requirement for prey 'knock-down'. Another singleton EST from the *E. c. sochureki *vgDbEST exhibited 81% identity to a kunitz-type protease inhibitor isolated from the elapid snake *Austrelaps labialis *[63]. Given the phylogenetic distance between these species, homology between these haemostatic disruptors is surprising, particularly since the singleton exhibited only 38% identity to kunitz-type protease inhibitors identified from the *Bitis gabonica *vgDbEST [38], a species closely related to *Echis*. An additional number of peptidases and purine liberators were identified as minor components in all but the *E. ocellatus *vgDbEST (Table 1). Despite their low representation and inconsistent conservation throughout the genus, the distinct biological activities of these components have been reported to play a role in the pathology of viper envenoming (Table 1), although these claims require experimental confirmation.

**Table 1:** Under-represented toxin encoding transcripts from the *Echis *vgDbESTs potentially associated with venom function.

Identification	No. of ESTs	Species present	Activty	Possible venom function
**Aminopeptidase**	8	*E. c. sochureki*	Hydrolysis of the N-terminal region of peptides [82].	Potential interference with angiogenesis and blood pressure control [83,84].
			
	1	*E. coloratus*		

**Ectonucletotide pyrophosphatase/phosphodiesterase**	2	*E. coloratus*	Hydrolysis of nucleotides and nucleic acids [85].	Interaction with platelet function [85]. Activity previously described in *Echis carinatus *[86].
			
	3	*E. c. sochureki*		

**5'-nucleotidase**	3	*E. coloratus*	Cleavage of a wide variety of ribose and deoxyribose nucleotides [1].	Potential inhibitor of platelet aggregation [1]. Activity identified in a number of different lineages including *Echis carinatus *[86].
			
	2	*E. p. leakeyi*		
			
	1	*E. c. sochureki*		

**Ectonucleoside triphosphate diphosphohydrolase 2 (E-NTPase 2)**	2	*E. coloratus*	Hydrolysis of nucleoside-5'-triphosphates and diphosphates [87].	Potential inhibitor of platelet aggregation [87,88].

### Novel venom gland transcriptome components

We identified a cluster from the *E. coloratus *vgDbEST that exhibited 64% identity to mammalian lysosomal acid lipase/cholesteryl ester hydrolase (LAL) [UniProt:Q4R4S5]. The most critical function of LAL is to modulate intracellular cholesterol metabolism by degrading cholesterol esters and triglycerides derived from low density lipoproteins that are transported, via specific receptors, into most cells [64,65]. Although LAL is a common enzyme in many lineages, this is the first time it has been identified from a venomous animal. We interrogated the vgDbESTs for other transcripts with annotations related to lysosomal processes and singleton transcripts were identified in multiple species (data not shown). However, their quantities were considerably lower than LAL suggesting to us that an association between venom gland LAL and intracellular processes was unlikely. Furthermore, the identification of a signal peptide using SignalP v3.0 [66] and the comparable representation of this enzyme (2%) with other venom toxin encoding transcripts (e.g. SPs, LAOs, growth factors), strongly implies these transcripts are a novel group of secreted venom components. Their biological contribution to the activity of *E. coloratus *venom and the venom gland and expression in other venomous snake genera is the subject of current research in our laboratories.

In addition to the discovery of LAL, two singleton transcripts were identified (additional file 2) from the *Echis *vgDbESTs as novel Serpentes zinc-dependent metallopeptidases [67]. A transcript exhibiting 67% identity to human dipeptidyl peptidase III (DPPIII) [UniProt:Q53GT4] was identified in *E. coloratus *and a related EST exhibiting 84% similarity to Neprilysin from *Gallus gallus *[Uniprot:Q67BJ2] was identified in the *E. p. leakeyi *vgDbEST. While signal peptides were absent from these ESTs due to EST N-terminal truncation, the constitutive physiological targets of their mammalian analogues indicate that these metallopeptidases may contribute to pathology. Mammalian DPPIII exhibits particular affinity for the degradation of hypertension-inducing peptides via the inactivation and degradation of angiotensin II to angiotensin III; the consequential reduction in vasoconstrictor activity likely induces hypotension alongside thrombolysis, by reducing the activity of plasminogen activator inhibitors that constrain fibrinolysis [68-70]. We previously reported that the *E. ocellatus *vgDbEST contained a substantial number of novel, potentially hypotensive, venom toxins termed the renin-like aspartic proteases [26]. Neprilysin demonstrates affinity for a broader range of physiological targets, including natriuretic, vasodilatory and neuro peptides [71]. Specific functional interactions include the termination of brain neuropeptides, such as enkephalins and substance P, at peptidergic synapses [72], and the degradation of the hypotension-inducing atrial natriuretic peptide (ANP) [71]. It is notable that Neprilysin has been implicated in the inactivation of peptide transmitters and their modulators in vertebrates and invertebrates [71,73], suggesting the potential for conserved neurotoxic activity across a range of prey species.

## Discussion

The most numerically abundant venom toxin families in the four *Echis *species were the SVMPs, CTLs, PLA_2_s, and SPs. This is broadly consistent with previous viperid venom gland analyses, although considerable inter-generic variations in the EST-inferred expression levels of these toxin families have been observed [26,38,52,53,56-59]. The correlation of toxin families identified from the genus *Echis *and other viperid species support current theories of early venom toxin recruitment prior to the radiation of the Viperidae [74]. The absence of three finger toxins from the *Echis *vgDbESTs is particularly notable as their recent identification in other viper species [53,58] implies the venom gland recruitment of these toxins occurred prior to the divergence of the Viperidae; presumably these toxins have subsequently been lost in an ancestor of *Echis*. Consistent with the early, PCR-driven, reports of accelerated evolution of venom serine proteases [75], CTLs [76] and PLA_2_s [77], it is apparent from the *Echis *genus vgDbESTs and those of other vipers that the evolutionary forces driving venom toxin recruitment in the genus *Echis *have served to promote diversification in some toxin lineages (PII and PIII SVMPs, CTLs) while in comparison relatively low diversification exists in others (PI and PIV SVMPs, PLA_2_s, LAOs, the growth factors, and remaining minor venom components). Prey capture is considered a major biological imperative driving the venom toxin selection process. This project was undertaken to identify correlations between intrageneric dietary preferences and transcript expression in order to elucidate the influence dietary selection pressures may have on the toxin composition of snake venoms.

(i) Recruitment of novel venom toxins and diet. The *Echis *vgDbESTs reveal the recruitment of novel renin-like aspartic proteases in *E. ocellatus *[26], LAL and DPPIII in *E. coloratus *and Neprilysin in *E. p. leakeyi*. The potential hypotensive role of venom aspartic proteases has been discussed previously [26]. Whilst expression in the venom proteome requires experimental verification, the presence of a signal peptide suggests that LAL is more likely to be secreted in the venom gland rather than acting as an intracellular protein. LAL has been implicated in severe alveolar destruction following over-expression of these enzymes in the lungs of mice [64]. Lipases such as LAL and lipoprotein lipase may also contribute to an influx of fatty acids into the brain by hydrolysing lipoproteins in the microvascular system of the cerebral cortex [78]. The suggestion that these fatty acids are then intra-cellularly internalised within lysosomes [78] correlates with intriguing observations from *E. coloratus *induced pathology, where increases in the size and numbers of lysosomes within the neuronal tissue of guinea pigs were implicated in neuron lysis and cerebral damage [79]. We infer from the predominately vertebrate-only diet of *E. coloratus *and the exclusive, yet substantial, representation of LAL in this species (2% - equivalent to the SPs, LAOs and growth factors) that LALs may play a contributory, albeit not yet understood, role in prey envenoming. As singletons, it is more difficult to argue that the novel recruitments of DPPIII and Neprilysin represent additional adaptations to prey preference; as they are found in such low numbers it is impossible to determine whether they are indeed novel species-specific venom gland recruitments or are rare transcripts that remain undetected in other snake species. We previously reported that invertebrate feeding likely evolved as a basal trait in the genus *Echis *[10]. The absence of genus-wide transcripts encoding novel putative venom toxin families implies that the adaptation to invertebrate feeding in *Echis *did not evolve as a consequence of recruiting novel invertebrate-specific venom toxins. However, we cannot exclude the possibility that the novel recruitment of LAL into the *E. coloratus *venom gland transcriptome may result from the subsequent reversion to vertebrate feeding observed in this species [10], particularly given the absence of these well represented putative toxin transcripts in other members of the genus.

(ii) Changes in toxin family expression and diet. All the major *Echis *venom toxin families (SVMP, CTL, PLA_2_, SP) exhibited considerable intrageneric variation in transcriptional representation. Thus, the *E. p. leakeyi *vgDbEST was notable for its absence of PI and PIV SVMPs, short coding disintegrins and CRISPs and atypically abundant representation of PII SVMPs, CTLs and PLA_2_s. The CRISPs were only represented by clusters in *E. c. sochureki *and *E. coloratus*, species whose vgDbESTs draw similarities, particularly in their high comparative expression of PIII SVMPs and short coding disintegrins. The only distinguishing feature (in terms of transcript abundance) in the *E. ocellatus *vgDbEST was the atypically high number of PIV SVMPs. However, none of these toxin encoding expression profiles showed a clear association with diet. Most notably, *E. p. leakeyi *and *E. c. sochureki *exhibit distinct toxin encoding profiles (Figure 2), despite both species feeding predominately on invertebrates and exhibiting highly invertebrate-lethal venom [10].

(iii) Diversification of venom toxins and diet. The above observations imply adaptations to diet are occurring within venom toxin families rather than resulting from changes in expression levels of entire toxin families. Evidence supporting this hypothesis is provided by substantial increases in representation of echicetin-like CTLs (relative to other CTLs) in both *E. p. leakeyi *and *E. c. sochureki*, implying perhaps a significant role for these platelet aggregation inhibitors in invertebrate prey capture. The absence of PI SVMPs in these species perhaps suggests that this SVMP isoform is more associated with a vertebrate diet. Furthermore, a number of atypical observations identified from the *E. coloratus *vgDbEST may be associated with a reversion to vertebrate feeding [10], including; (i) increases in the representation of CRISPs, (ii) increases in cluster diversity of the SPs and (iii) the identification of putative novel venom toxins (LAL and DPPIII). However, the general similarity between the toxin encoding expression profiles of *E. c. sochureki *and *E. coloratus *(Figure 2), despite *E. coloratus *exhibiting a significant reduction in venom toxicity to invertebrates [10], indicates that more analytical molecular tools are required to determine whether snake prey specificity is achieved through subtle alterations in isoform expression levels within the major venom toxin families. We are subjecting the *Echis *genus vgDbEST data generated here to a phylogenetic analysis on each toxin class to determine species-specific trends in diversification, which will inform us whether multiple levels of gene control in the *Echis *genus venom gland (switching of transcriptional expression, gene duplication conferring functional diversification and novel gene expression) maybe responsible for evolutionary responses to dietary pressures.

Correlations between variation in venom gland toxin encoding profiles and snakebite symptomatologies from the genus *Echis *are unclear, particularly given the similar, predominately incoagulable and haemorrhagic, clinical outcomes observed throughout the genus [19-21] and the presence of multiple isoforms of toxin families implicated in haemorrhage and coagulopathy. However, some observations of atypical symptoms can be tentatively explained; substantial increases in PLA_2 _representation and the unique presence of Neprilysin may correlate with the rare manifestation of neurotoxicity observed in an *E. pyramidum *envenomation [22], whilst the putative function of DPPIII may imply a contributory role in cases of hypotension observed following *E. coloratus *snakebite [20].

Venom gland transcriptome surveys provide valuable new data that we are correlating with a proteomic analysis of the venom from each *Echis *species. With this comprehensive description of the venom composition of each major *Echis *lineage, we will identify, using proteomic (antivenomic) techniques [3], the extent to which the intrageneric variation in venom composition impacts on the preclinical efficacy of commercially available antivenoms. We hope that such analyses will (i) explain past antivenom failures described following snakebite by members of this medically important genus [18,22-24] and (ii) identify the venom toxin mix required to generate an antivenom with continent-wide clinical effectiveness against *Echis *envenoming.

## Conclusion

The first comprehensive comparison of intrageneric venom gland transcriptomes reveals substantial venom variation in the genus *Echis*. The observed variations in venom toxin encoding profiles reveal little association with venom adaptations to diet previously described from this genus. We hypothesise that relatively subtle alterations in toxin expression levels within the major venom toxin families are likely to be predominately responsible for prey specificity, although we cannot rule out a contributory role for novel putative venom toxins, such as lysosomal acid lipase. The observation of substantial venom variation within the medically important genus *Echis *strongly advocates further investigations into the medical significance of venom variation and its potential impact upon antivenom therapy.

## Methods

Venom gland cDNA libraries were constructed from ten wild-caught specimens of *Echis coloratus *(Egypt), *E. p. leakeyi *(Kenya)and *E. c. sochureki *(Sharjah, UAE), maintained in the herpetarium of the Liverpool School of Tropical Medicine, using identical protocols described for the construction of the venom gland cDNA library from *E. ocellatus *[26]. Clones from the cDNA libraries were picked randomly and sequenced (NERC Molecular Genetics Facility, UK) using M13 forward primers.

Bioinformatic processing was carried out using the PartiGene pipeline [80] with the same protocols used previously [26]. Briefly, sequences were processed (to exclude low quality, contaminating vector sequences and poly A+ tracts) using Trace2dbEST [81]. Subsequently, assembly was undertaken in PartiGene version 3.0, using high stringency clustering parameters [26,81]. A total of 1070 (*E. coloratus*), 1078 (*E. p. leakeyi*) and 1156 (*E. c. sochureki*) processed ESTs were entered into respective species databases alongside the 883 ESTs generated from the *E. ocellatus *vgDbEST [26]. Assembled ESTs were BLAST annotated against UniProt (v56.2), TrEMBL (v39.2) and separate databases containing only Serpentes nucleotide and protein sequences derived from the same Uniprot/TrEMBL release versions.

Clustering was performed incrementally (96 sequences per round) to determine the number of sequences required to construct a representative transcriptome (i.e. the point where further sequencing only adds to existing clusters). We estimate that a minimum of 800 EST sequences were required to provide an accurate representation of the three vgDbESTs (additional file 3). For longer clones (i.e. SVMPs), representatives of each cluster were subject to primer walking to acquire sufficient sequence data for isoform classification. SVMPs were characterised based upon the presence or absence of additional domains extending from the metalloproteinase domain [30]. PIVs were distinguished from PIIIs by the presence of an additional cysteine residue in the cysteine-rich region at positions 397 or 400 [[28,30] (numbering from 30)].

Additional file 2 displays the catalogue of venom toxin transcripts present in each of the four *Echis *vgDbESTs based upon significant (>1e-05) BLAST annotation. Presentation of the fully assembled and annotated vgDbESTs can be viewed at http://venoms.liv.ac.uk. The sequences reported in this paper have also been submitted into dbEST division of the public database GenBank: *E. coloratus *[GenBank: GR947900-GR948969], *E. c. sochureki *[GenBank: GR948970-GR950126] and *E. p. leakeyi *[GenBank: GR950127-GR951204].

All animal experimentation was conducted using standard protocols approved by the University of Liverpool Animal Welfare Committee and performed with the approval of the UK Home Office (40/3216) under project licence # 40/3216.

## Authors' contributions

NRC participated in the experiments, the comparative analysis and drafted the manuscript. RAH participated in the experiments, the design of the study and reviewed the manuscript. WW participated in the design of the study and reviewed the manuscript. SCW participated in the experiments, the design of the study, the comparative analysis and reviewed the manuscript. All authors have read and approved the paper.

## Supplementary Material

Additional file 1 Summary statistics following clustering and assembling of ESTs for *E. coloratus*, *E. p. leakeyi *and *E. c. sochureki*. Click here for file

Additional file 2 **Catalogue of venom toxin encoding ESTs determined from the *Echis *vgDbESTs**. Putative novel venom toxins are in bold and underlined. Key - SVMP: snake venom metalloproteinases; PI, PII, PIII, PIV: respective sub-group of SVMPs; ND: sub-class not determined; DIS: short coding disintegrins; CTL: C-type lectins; PLA_2_: group II phospholipases A_2_; SP: serine proteases; LAO: L-amino oxidases; CRISP: cysteine-rich secretory proteins; VEGF: vascular endothelial growth factors; NGF: nerve growth factors; PEPT: peptidases; AP: aminopeptidase; DPP: dipeptidyl peptidase III; NEP: neprilysin; PE: Purine liberators; PHOS: phosphdiesterase; 5'-NUC: 5'-nucleotidase; E-NTPase: ectonucleoside triphosphate diphosphohydrolase; LAL: lysosomal acid lipases; RLAP: renin-like aspartic proteases; HYAL: hyaluronidases; KTZ: kunitz-type protease inhibitors. Click here for file

Additional file 3 **An overview of clustering processes for three species of the genus *Echis***. The graph demonstrates the percentage of ESTs that are added to clusters (ESTs >1) as the cumulative number of ESTs entering the database increase. In all species the number of ESTs affecting the proportion of EST clusters and singletons reaches a plateau after 800 sequences. Click here for file
