# Supplementary material for: Comparative venom gland transcriptome surveys of the saw-scaled vipers (Viperidae: Echis) reveal substantial intra-family gene diversity and novel venom transcripts
Source: BMC Genomics. 2009 Nov 30;10:564. doi: 10.1186/1471-2164-10-564 (PMC2790475; doi:10.1186/1471-2164-10-564)
Supplement: Additional file 1 — Summary statistics following clustering and assembling of ESTs for E. coloratus, E. p. leakeyi and E. c. sochureki. [file 1471-2164-10-564-S1.doc]

**Additional file 1. Summary statistics following clustering and assembling of ESTs for *E. coloratus*, *E. p. leakeyi* and *E. c. sochureki*.**

|  | ***E. coloratus*** | | | ***E. p. leakeyi*** | | | ***E. c. sochureki*** | | |
| --- | --- | --- | --- | --- | --- | --- | --- | --- | --- |
|  | No. of clusters | No. of ESTs | % of ESTs | No. of clusters | No. of ESTs | % of ESTs | No. of clusters | No. of ESTs | % of ESTs |
| **Clusters >1** |  |  |  |  |  |  |  |  |  |
| **- Toxin** | 62 | 612 | 57.20 | 50 | 717 | 66.51 | 54 | 502 | 43.39 |
| **- Non-toxin** | 36 | 135 | 12.62 | 18 | 84 | 7.79 | 39 | 209 | 18.06 |
| **- Unidentified** | 2 | 5 | 0.47 | 4 | 8 | 0.74 | 7 | 26 | 2.25 |
| **Singletons** |  |  |  |  |  |  |  |  |  |
| **- Toxin** | - | 50 | 4.67 | - | 42 | 3.90 | - | 42 | 3.63 |
| **- Non-toxin** | - | 196 | 18.31 | - | 121 | 11.23 | - | 182 | 15.73 |
| **- Unidentified** | - | 72 | 6.73 | - | 106 | 9.83 | - | 196 | 16.94 |
|  |  |  |  |  |  |  |  |  |  |
| **Totals** | 100 | 1070 | 100 | 72 | 1078 | 100 | 100 | 1157 | 100 |
