# Supplementary material for: Comparative venom gland transcriptome surveys of the saw-scaled vipers (Viperidae: Echis) reveal substantial intra-family gene diversity and novel venom transcripts
Source: BMC Genomics. 2009 Nov 30;10:564. doi: 10.1186/1471-2164-10-564 (PMC2790475; doi:10.1186/1471-2164-10-564)
Supplement: Additional file 2 — Catalogue of venom toxin encoding ESTs determined from the Echis vgDbESTs. Putative novel venom toxins are in bold and underlined. Key - SVMP: snake venom metalloproteinases; PI, PII, PIII, PIV: respective sub-group of SVMPs; ND: sub-class not determined; DIS: short coding disintegrins; CTL: C-type lectins; PLA2: group II phospholipases A2; SP: serine proteases; LAO: L-amino oxidases; CRISP: cysteine-rich secretory proteins; VEGF: vascular endothelial growth factors; NGF: nerve growth factors; PEPT: peptidases; AP: aminopeptidase; DPP: dipeptidyl peptidase III; NEP: neprilysin; PE: Purine liberators; PHOS: phosphdiesterase; 5'-NUC: 5'-nucleotidase; E-NTPase: ectonucleoside triphosphate diphosphohydrolase; LAL: lysosomal acid lipases; RLAP: renin-like aspartic proteases; HYAL: hyaluronidases; KTZ: kunitz-type protease inhibitors. [file 1471-2164-10-564-S2.doc]

**Additional file 2. Catalogue of venom toxin encoding ESTs determined from the *Echis* vgDbESTs.**

| Venom toxin family | ***E. coloratus*** | | ***E. p. leakeyi*** | | ***E. ocellatus*** | | | ***E. c. sochureki*** | | |
| --- | --- | --- | --- | --- | --- | --- | --- | --- | --- | --- |
|  | Cluster ID | ESTs/ cluster | Cluster ID | ESTs/ cluster | Cluster ID | | ESTs/ cluster | Cluster ID | | ESTs/ cluster |
| **SVMP** |  |  |  |  |  | |  |  | |  |
| **Class PI** | ECO00047 | 10 | None | - | EOC00028 | | 21 | None | | - |
|  |  |  |  |  | EOC00004 | | 4 |  | |  |
| Total ESTs |  | 10 |  | 0 |  | | 25 |  | | 0 |
| **Class PII** | ECO00011 | 60 | EPL00005 | 134 | EOC00006 | | 20 | ECS00117 | | 20 |
|  | ECO00020 | 38 | EPL00006 | 91 | EOC00071 | | 12 | ECS00012_2 | | 19 |
|  | ECO00017 | 27 | EPL00056 | 10 |  | |  | ECS00114 | | 11 |
|  | ECO00027 | 17 | EPL00097 | 9 |  | |  | ECS00253 | | 8 |
|  | ECO00044 | 4 |  |  |  | |  | ECS00059 | | 3 |
|  |  |  |  |  |  | |  | ECS00086 | | 3 |
| Total ESTs |  | 146 |  | 244 |  | | 32 |  | | 64 |
| **Class PIII** | ECO00002 | 42 | EPL00008 | 25 | EOC00063 | | 22 | ECS00012_1 | | 52 |
|  | ECO00007 | 26 | EPL00004 | 22 | EOC00013 | | 11 | ECS00053 | | 42 |
|  | ECO00023 | 22 | EPL00002 | 12 | EOC00001 | | 9 | ECS00031 | | 30 |
|  | ECO00012 | 20 | EPL00090 | 6 | EOC00089 | | 9 | ECS00062 | | 19 |
|  | ECO00010 | 18 | EPL00040 | 5 | EOC00008 | | 6 | ECS00257 | | 11 |
|  | ECO00009 | 16 | EPL00029 | 4 | EOC00081 | | 6 | ECS00071 | | 9 |
|  | ECO00067 | 14 | EPL00061 | 4 | EOC00086 | | 5 | ECS00003 | | 6 |
|  | ECO00050 | 9 | EPL00125 | 4 | EOC00095 | | 5 | ECS00030 | | 6 |
|  | ECO00001 | 7 | EPL00019 | 3 | EOC00186 | | 4 | ECS00044 | | 6 |
|  | ECO00034 | 7 | EPL00032 | 3 | EOC00073 | | 3 | ECS00056 | | 4 |
|  | ECO00004 | 6 | EPL00044 | 3 | EOC00016 | | 3 | ECS00163 | | 4 |
|  | ECO00106 | 5 | EPL00055 | 3 | EOC00404 | | 3 | ECS00177 | | 4 |
|  | ECO00275 | 5 | EPL00103 | 3 | EOC00016 | | 3 | ECS00043 | | 3 |
|  | ECO00076 | 4 | EPL00159 | 2 | EOC00404 | | 3 | ECS00120 | | 3 |
|  | ECO00406 | 3 | EPL00396 | 2 |  | |  | ECS00251 | | 3 |
|  | ECO00146 | 2 |  |  |  | |  | ECS00213 | | 2 |
|  | ECO00192 | 2 |  |  |  | |  | ECS00456 | | 2 |
|  | ECO00222 | 2 |  |  |  | |  | ECS00497 | | 2 |
|  |  |  |  |  |  | |  | ECS00678 | | 2 |
| Total ESTs |  | 210 |  | 101 |  | | 84 |  | | 210 |
| **Class PIV** | ECO00144 | 7 | None | - | EOC00024 | | 55 | ECS00087 | | 10 |
|  | ECO00061 | 2 |  |  | EOC00022 | | 17 |  | |  |
|  | ECO00075 | 2 |  |  |  | |  |  | |  |
| Total ESTs |  | 11 |  | 0 |  | | 72 |  | | 10 |
|  |  |  |  |  |  | |  |  | |  |
| **ND and singletons** |  | 28 |  | 33 |  | | 27 |  | | 29 |
| **DIS** | ECO00024 | 36 | Singletons | 1 | None | | - | ECS00035 | | 20 |
|  | Singletons | 1 |  |  |  | |  | ECS00036 | | 19 |
|  |  |  |  |  |  | |  | Singletons | | 1 |
| Total ESTs |  | 37 |  | 1 |  | | 0 |  | | 40 |
|  |  |  |  |  |  | |  |  | |  |
| Venom toxin family | ***E. coloratus*** | | ***E. p. leakeyi*** | | ***E. ocellatus*** | | | ***E. c. sochureki*** | | |
|  | Cluster ID | ESTs/ cluster | Cluster ID | ESTs/ cluster | Cluster ID | ESTs/ cluster | | Cluster ID | ESTs/ cluster | |
| **CTL** | ECO00038 | 19 | EPL00010 | 39 | EOC00124 | 6 | | ECS00050 | 14 | |
|  | ECO00127 | 10 | EPL00066 | 27 | EOC00125 | 3 | | ECS00102 | 13 | |
|  | ECO00069 | 5 | EPL00016 | 21 | EOC00133 | 3 | | ECS00154 | 11 | |
|  | ECO00108 | 5 | EPL00038 | 16 | EOC00334 | 3 | | ECS00230 | 10 | |
|  | ECO00070 | 4 | EPL00031 | 13 | EOC00083 | 2 | | ECS00098 | 8 | |
|  | ECO00052 | 3 | EPL00030 | 9 | EOC00092 | 2 | | ECS00006 | 7 | |
|  | ECO00041 | 2 | EPL00053 | 9 | Singletons | 18 | | ECS00045 | 7 | |
|  | ECO00115 | 2 | EPL00109 | 8 |  |  | | ECS00038 | 6 | |
|  | ECO00153 | 2 | EPL00034 | 6 |  |  | | ECS00140 | 3 | |
|  | ECO00158 | 2 | EPL00112 | 6 |  |  | | ECS00051 | 2 | |
|  | ECO00197 | 2 | EPL00081 | 5 |  |  | | ECS00346 | 2 | |
|  | ECO00270 | 2 | EPL00018 | 3 |  |  | | Singletons | 8 | |
|  | Singletons | 10 | EPL00127 | 3 |  |  | |  |  | |
|  |  |  | EPL00060 | 2 |  |  | |  |  | |
|  |  |  | EPL00078 | 2 |  |  | |  |  | |
|  |  |  | EPL00282 | 2 |  |  | |  |  | |
|  |  |  | Singletons | 11 |  |  | |  |  | |
| Total ESTs |  | 68 |  | 182 |  | 37 | |  | 91 | |
| **PLA_2_** |  |  |  |  |  |  | |  |  | |
| **Asp^49^** | ECO00086 | 11 | EPL00071 | 51 | EOC00079 | 10 | | ECS00002 | 17 | |
|  | ECO00186 | 3 | EPL00001 | 33 |  |  | |  |  | |
|  |  |  | EPL00204 | 2 |  |  | |  |  | |
|  |  |  |  |  |  |  | |  |  | |
| **Ser^49^** | ECO00035 | 21 | EPL00012 | 52 | EOC00015 | 15 | | ECS00014 | 23 | |
|  |  |  | EPL00195 | 11 |  |  | |  |  | |
|  |  |  |  |  |  |  | |  |  | |
| **ND** | None | - | EPL00274 | 3 | Singletons | 4 | | Singletons | 3 | |
|  |  |  | Singletons | 4 |  |  | |  |  | |
| Total ESTs |  | 35 |  | 156 |  | 29 | |  | 43 | |
| **SP** | ECO00285 | 4 | EPL00089 | 6 | EOC00049 | 5 | | ECS00244 | 11 | |
|  | ECO00013 | 3 | EPL00098 | 2 | Singletons | 3 | | ECS00134 | 5 | |
|  | ECO00112 | 3 | EPL00435 | 2 |  |  | | ECS00186 | 4 | |
|  | ECO00117 | 2 | Singletons | 5 |  |  | | ECS00105 | 3 | |
|  | ECO00119 | 2 |  |  |  |  | | Singletons | 2 | |
|  | ECO00135 | 2 |  |  |  |  | |  |  | |
|  | ECO00164 | 2 |  |  |  |  | |  |  | |
|  | ECO00182 | 2 |  |  |  |  | |  |  | |
|  | ECO00419 | 2 |  |  |  |  | |  |  | |
|  | Singletons | 7 |  |  |  |  | |  |  | |
| Total ESTs |  | 29 |  | 15 |  | 8 | |  | 25 | |
| **LAO** | ECO00026 | 24 | EPL00025 | 19 | EOC00167 | 2 | | ECS00178 | 4 | |
|  | Singletons | 2 | Singletons | 1 | EOC00233 | 2 | | ECS00061 | 2 | |
| Total ESTs |  | 26 |  | 20 |  | 4 | |  | 6 | |
| **CRISP** | ECO00025 | 33 | None | - | Singletons | 1 | | ECS00093 | 4 | |
|  | Singletons | 2 |  |  |  |  | | ECS00169 | 6 | |
| Total ESTs |  | 35 |  | 0 |  | 1 | |  | 10 | |
|  |  |  |  |  |  |  | |  |  | |
| Venom toxin family | ***E. coloratus*** | | ***E. p. leakeyi*** | | ***E. ocellatus*** | | | ***E. c. sochureki*** | | |
|  | Cluster ID | ESTs/ cluster | Cluster ID | ESTs/ cluster | Cluster ID | ESTs/ cluster | | Cluster ID | ESTs/ cluster | |
| **VEGF** | ECO00199 | 2 | EPL00139 | 2 | EOC00176 | 6 | | ECS00431 | 2 | |
|  |  |  |  |  | EOC00478 | 2 | |  |  | |
|  |  |  |  |  |  |  | |  |  | |
| **NGF** | ECO00049 | 2 | EPL00043 | 2 | Singletons | 1 | | Singletons | 1 | |
|  |  |  |  |  |  |  | |  |  | |
| **PEPT** |  |  |  |  |  |  | |  |  | |
| **AP** | Singletons | 1 | None | - | None | - | | ECS00179 | 7 | |
|  |  |  |  |  |  |  | | Singletons | 1 | |
| **DPP** | Singletons | **1** | None | - | None | - | | None | - | |
| **NEP** | None | - | Singletons | **1** | None | - | | None | - | |
|  |  |  |  |  |  |  | |  |  | |
| **PE** |  |  |  |  |  |  | |  |  | |
| **PHOS** | ECO00241 | 2 | None | - | None | - | | ECS00101 | 2 | |
|  |  |  |  |  |  |  | | Singletons | 1 | |
| **5’-NUC** | ECO00276 | 2 | Singletons | 2 | None | - | | Singletons | 1 | |
|  | Singletons | 1 |  |  |  |  | |  |  | |
|  |  |  |  |  |  |  | |  |  | |
| **E-NTPase** | ECO00014 | 2 | None | - | None | - | | None | - | |
|  |  |  |  |  |  |  | |  |  | |
| **LAL** | ECO00073 | **13** | None | - | None | - | | None | - | |
|  | Singletons | **1** |  |  |  |  | |  |  | |
|  |  |  |  |  |  |  | |  |  | |
|  |  |  |  |  |  |  | |  |  | |
| **RLAP** | None | - | None | - | EOC00051 | **10** | | None | - | |
|  |  |  |  |  | EOC00123 | **4** | |  |  | |
|  |  |  |  |  | Singletons | **3** | |  |  | |
|  |  |  |  |  |  |  | |  |  | |
| **HYAL** | None | - | None | - | Singletons | 1 | | Singletons | 1 | |
|  |  |  |  |  |  |  | |  |  | |
| **KTZ** | None | - | None | - | None | - | | Singletons | 1 | |
|  |  |  |  |  |  |  | |  |  | |

Putative novel venom toxins are in bold and underlined. Key – SVMP: snake venom metalloproteinases; PI, PII, PIII, PIV: respective sub-group of SVMPs; ND: sub-class not determined; DIS: short coding disintegrins; CTL: C-type lectins; PLA_2_: group II phospholipases A_2_; SP: serine proteases; LAO: L-amino oxidases; CRISP: cysteine-rich secretory proteins; VEGF: vascular endothelial growth factors; NGF: nerve growth factors; PEPT: peptidases; AP: aminopeptidase; DPP: dipeptidyl peptidase III; NEP: neprilysin; PE: Purine liberators; PHOS: phosphdiesterase; 5’-NUC: 5’-nucleotidase; E-NTPase: Ectonucleoside triphosphate diphosphohydrolase; LAL: lysosomal acid lipases; RLAP: renin-like aspartic proteases; HYAL: hyaluronidases; KTZ: kunitz-type protease inhibitors.
