# Supplementary figures and images for: Comparative venom gland transcriptome surveys of the saw-scaled vipers (Viperidae: Echis) reveal substantial intra-family gene diversity and novel venom transcripts
Source: BMC Genomics. 2009 Nov 30;10:564. doi: 10.1186/1471-2164-10-564 (PMC2790475; doi:10.1186/1471-2164-10-564)

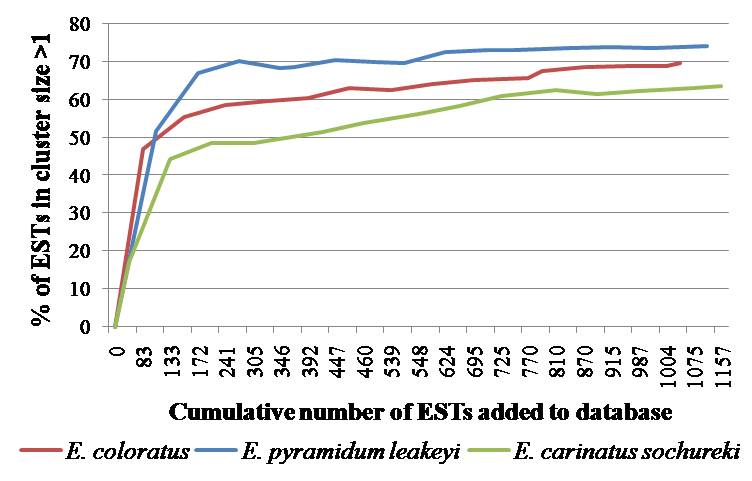

Supplement: Additional file 3 — An overview of clustering processes for three species of the genus Echis. The graph demonstrates the percentage of ESTs that are added to clusters (ESTs >1) as the cumulative number of ESTs entering the database increase. In all species the number of ESTs affecting the proportion of EST clusters and singletons reaches a plateau after 800 sequences. [file 1471-2164-10-564-S3.jpeg]
